# Arrhythmogenic Risk in iPSC-Derived Cardiomyocytes: Current Limitations and Therapeutic Perspectives

**DOI:** 10.3390/medicina61112056

**Published:** 2025-11-18

**Authors:** Dhienda C. Shahannaz, Tadahisa Sugiura, Brandon E. Ferrell, Taizo Yoshida

**Affiliations:** 1 Faculty of Medicine, Universitas Indonesia, Jakarta 10430, Indonesia; dhiendaladdynasrul@gmail.com; 2 Department of Cardiothoracic and Vascular Surgery, Montefiore Medical Center/Albert Einstein College of Medicine, Bronx, NY 10467, USA; 3 Department of General Surgery, Montefiore Medical Center/Albert Einstein College of Medicine, Bronx, NY 10467, USA

**Keywords:** iPSC-derived cardiomyocytes, arrhythmogenic risk, cardiac arrhythmias, stem cell therapy, electrophysiological modeling, proarrhythmic potential, gene editing in cardiology, cardiac maturation protocols, co-culture systems in cardiomyocytes, pharmacological interventions in arrhythmia

## Abstract

*Background and Objectives*: Induced pluripotent stem cell-derived cardiomyocytes (iPSC-CMs) have revolutionized cardiac research by providing patient-specific models for studying arrhythmias. However, their clinical application is hindered by arrhythmogenic risks associated with grafted iPSC-CMs. This review aims to delineate the current limitations in iPSC-CM-based arrhythmia modeling and explore emerging therapeutic strategies to mitigate these risks. *Materials and Methods*: A comprehensive literature review was conducted, focusing on studies published in the last two decades that address the electrophysiological characteristics of iPSC-CMs, their arrhythmogenic potential, and therapeutic interventions. Sources include peer-reviewed journals, clinical trial reports, and recent advancements in stem cell technology. *Results*: Findings indicate that while iPSC-CMs offer a promising platform for arrhythmia modeling, challenges such as cellular heterogeneity, immaturity, and proarrhythmic potential persist. Advancements in maturation protocols, co-culture systems, and gene editing techniques have shown promise in enhancing the safety profile of iPSC-CMs. *Conclusions*: Addressing the arrhythmogenic risks associated with iPSC-CMs requires a multifaceted approach, including improved differentiation protocols, maturation strategies, and therapeutic interventions. Continued research is essential to translate these models into safe and effective clinical applications.

## 1. Introduction

Induced pluripotent stem cells (iPSCs) have transformed cardiovascular research by providing a renewable source of patient-specific cardiomyocytes [1,2,3]. Through reprogramming of somatic cells, iPSCs can be differentiated into cardiomyocytes (iPSC-CMs) that retain the donor’s genetic background [1,3], enabling faithful modeling of inherited cardiac disorders. This breakthrough bypasses the limitations of primary human cardiomyocytes [1,3], which are difficult to obtain and expand, and animal models, which often fail to capture human-specific disease mechanisms [2,3,4]. iPSC-CMs have therefore become an indispensable tool for disease modeling, pharmacological testing, and regenerative applications.

In the field of arrhythmia research, iPSC-CMs offer a uniquely powerful platform. Because arrhythmias frequently arise from genetic mutations in ion channels or structural proteins [1,3], patient-derived iPSC-CMs allow direct investigation of electrophysiological abnormalities at the cellular level [1,3,5]. Multi-electrode array (MEA) and patch-clamp studies have shown that iPSC-CMs recapitulate arrhythmogenic phenotypes such as prolonged action potential duration, irregular beating, and triggered activity [1,5,6,7,8,9]. They have been instrumental in advancing our understanding of long QT syndrome [10], catecholaminergic polymorphic ventricular tachycardia [11,12], Brugada syndrome [13], and atrial fibrillation [14]. Moreover, the capacity of iPSC-CMs to integrate into engineered tissues or 3D models has provided a bridge between cellular pathology and whole-heart physiology [1,2,3]. This aligns with emerging regenerative strategies that seek to repair damaged myocardium with cell-based therapies, a theme recurrent across recent work on mitochondrial dynamics, extracellular vesicles, and bioenergetics restoration [15,16,17].

Despite these advances, the clinical and translational use of iPSC-CMs remains constrained by arrhythmogenic risks. Their relative immaturity, heterogeneity, and incomplete electrophysiological fidelity increase the likelihood of proarrhythmic events. Furthermore, grafting iPSC-CMs into injured myocardium introduces risks of ectopic foci [18], conduction block [19], and electrical instability [1,3]. As highlighted in recent investigations of stem cell therapies for heart failure and the mechanistic role of extracellular vesicles in cardioprotection, safety remains the decisive barrier to therapeutic implementation.

The present review critically evaluates the arrhythmogenic risks of iPSC-derived cardiomyocytes and synthesizes therapeutic perspectives aimed at mitigating these limitations. Specifically, it examines advances in maturation protocols [3,15,16,17], genetic and epigenetic editing [15,16,17], engineered tissue integration [2,3,15,16], and pharmacological modulation [20]. By integrating insights from disease modeling, bioenergetics-focused interventions, and regenerative cardiology, this review aims to define a path toward safer and more effective use of iPSC-CMs in both preclinical and clinical contexts.

## 2. Materials and Methods

### 2.1. Literature Search Strategy

A comprehensive literature search was conducted between January 2004 and August 2025 to identify studies examining the arrhythmogenic properties, electrophysiological characteristics, and therapeutic mitigation strategies of induced pluripotent stem cell-derived cardiomyocytes (iPSC-CMs). The databases PubMed, Scopus, Web of Science, and Google Scholar were systematically queried using Boolean combinations of key terms, including “iPSC-derived cardiomyocytes”, “arrhythmia”, “electrophysiology”, “immaturity”, “mitochondria”, “heterogeneity”, “gene editing”, “maturation protocols”, “co-culture”, and “pharmacological modulation.”

To optimize discoverability and align with recent citation trends, additional searches were performed in MDPI, Frontiers, Nature Communications, and Elsevier databases using similar keywords and MeSH term expansions such as “cardiac differentiation,” “excitation-contraction coupling,” and “regenerative cardiology.” Reference lists of retrieved papers and relevant review articles were manually screened to identify additional studies that fulfilled the inclusion criteria.

### 2.2. Selection Criteria

Studies were included if they met one or more of the following criteria:

(1) experimental research involving human or animal iPSC-CMs addressing electrophysiological, structural, or metabolic correlates of arrhythmogenicity;
(2) interventional studies evaluating strategies to mitigate arrhythmic risk through maturation protocols, genetic or epigenetic editing, co-culture systems, or pharmacological modulation; and
(3) reviews or meta-analyses that provide mechanistic or translational insights into iPSC-CM electrophysiology and bioenergetics.

Publications were limited to English-language articles spanning January 2004–August 2025, reflecting the period from the seminal discovery of iPSCs to the present era of translational maturation and safety optimization. Exclusion criteria included non-cardiac iPSC models, non-peer-reviewed sources (e.g., conference abstracts, theses), studies lacking electrophysiological data, and reports focused exclusively on pluripotency or unrelated differentiation lineages.

### 2.3. Data Analysis

Data were synthesized using an integrative qualitative approach, emphasizing the convergence of electrophysiological, metabolic, and structural determinants of arrhythmogenicity. Each study was evaluated for its contribution to three central themes: (1) mechanistic basis of proarrhythmic phenotypes in iPSC-CMs, (2) experimental and translational mitigation strategies, and (3) relevance to clinical safety and regenerative applications. Quantitative findings, where available, such as changes in action potential duration, ion current density, or conduction velocity, were qualitatively summarized rather than meta-analyzed due to methodological heterogeneity across studies.

To ensure conceptual consistency, cross-validation of data interpretation was performed by aligning the extracted evidence with previously published works from our group on mitochondrial bioenergetics, extracellular vesicle modulation, and regenerative cardiac electrophysiology [1,2,3,5,15,16,17]. This triangulated synthesis allowed for the construction of a multidimensional model of arrhythmogenic risk, integrating electrophysiological, cellular, and bioenergetic domains to inform therapeutic perspectives and figure conceptualization.

## 3. Arrhythmogenic Risks in iPSC-CMs

### 3.1. Electrophysiological Characteristics

The arrhythmogenic profile of induced pluripotent stem cell-derived cardiomyocytes (iPSC-CMs) is fundamentally rooted in their electrophysiological phenotype, which remains distinct from that of adult ventricular cardiomyocytes [3,5,15]. iPSC-CMs typically display spontaneous automaticity, reflecting pacemaker-like behavior due to incomplete suppression of HCN channel activity and elevated funny current (I_f) density [21,22,23]. Their action potential duration (APD) is generally prolonged and highly variable [3,5], driven by reduced expression of the inward rectifier potassium channel (IK1; encoded by KCNJ2), resulting in depolarized resting membrane potentials [5,24,25,26]. Additionally, calcium handling abnormalities are frequent [1,3,5], as immature sarcoplasmic reticulum function and reduced expression of ryanodine receptor 2 (RyR2) impair calcium-induced calcium release [5,27,28,29] thereby promoting delayed afterdepolarizations (DADs) [29,30].

In disease-specific iPSC-CM models, these electrophysiological discrepancies amplify disease phenotypes. Long QT syndrome iPSC-CMs display abnormal repolarization kinetics due to altered IKr or IKs currents [31,32] while catecholaminergic polymorphic ventricular tachycardia models reproduce abnormal Ca^2+^ transients under adrenergic stimulation [33]. These insights parallel our own observations in regenerative paradigms, where energy metabolism directly intersects with electrophysiology: mitochondrial dysfunction or altered redox signaling can destabilize action potentials, a theme we highlighted in our recent work on mitochondrial dynamics and extracellular vesicle-based therapies [1,16].

To contextualize these electrophysiological signatures across the literature, Table 1 summarizes key 2D iPSC-CM models of inherited and acquired cardiac arrhythmias, detailing their genetic backgrounds, experimental platforms, and major electrophysiological findings. This comparative overview highlights how immature ionic profiles and metabolic underpinnings converge to reproduce clinical arrhythmia phenotypes in vitro while emphasizing the need for standardization in future regenerative applications (Table 1).

Taken together, the incomplete recapitulation of adult ion channel expression and excitation-contraction coupling renders iPSC-CMs inherently susceptible to proarrhythmic events, both in vitro and when considered for transplantation into host myocardium.

### 3.2. Cellular Heterogeneity

Another critical determinant of arrhythmogenic risk in iPSC-CMs is cellular heterogeneity, which manifests at both population and tissue scales. Differentiation protocols routinely generate a mixed pool of ventricular-, atrial-, and nodal-like phenotypes, as revealed by action potential morphology and subtype-specific marker expression [3,57,58]. Such heterogeneity, while reflective of early developmental cardiac biology, introduces electrical dispersion and anisotropic conduction when these cells are integrated into engineered tissues [59,60]. Local conduction heterogeneity fosters reentrant circuits, a substrate for sustained arrhythmias [61,62,63].

Furthermore, within subpopulations of ventricular-like cells, stochastic variability in ion channel gene expression and metabolic state drives beat-to-beat instability [64,65,66,67,68,69]. Stem-cell derived models of heart failure emphasized that these metabolic disparities—particularly differences in mitochondrial network integrity and substrate utilization—exacerbate proarrhythmic vulnerability [1,2]. Similarly, in mitochondria-enriched extracellular vesicles, it is noted that variability in donor vesicle content translates into unequal rescue of energetic function across recipient cardiomyocytes, underscoring the biological consequences of heterogeneity at the subcellular and paracrine levels [5].

At systems perspective, heterogeneity is double-edged: it enables versatile disease modeling across arrhythmic syndromes, yet in regenerative applications it elevates the likelihood of conduction block, ectopy, and fibrillatory dynamics [19,70,71,72]. Addressing this will require both upstream improvements in directed differentiation and downstream engineering solutions to enforce structural alignment and subtype purity.

### 3.3. Immaturity of iPSC-CMs

Perhaps the most pervasive limitation of iPSC-CMs is their developmental immaturity, which drives much of their arrhythmogenicity. Morphologically, iPSC-CMs exhibit small size, disorganized sarcomeres, and underdeveloped T-tubule networks [5,15,16,73]. Functionally, they rely predominantly on glycolysis rather than oxidative phosphorylation [1,5,15,16], leading to mismatched bioenergetics under high workload [5,15,16]. Mitochondrial cristae are sparse and fragmented [5], limiting calcium buffering and ATP delivery to contractile machinery [5]—an energetic substrate of proarrhythmia.

**Figure 1:**
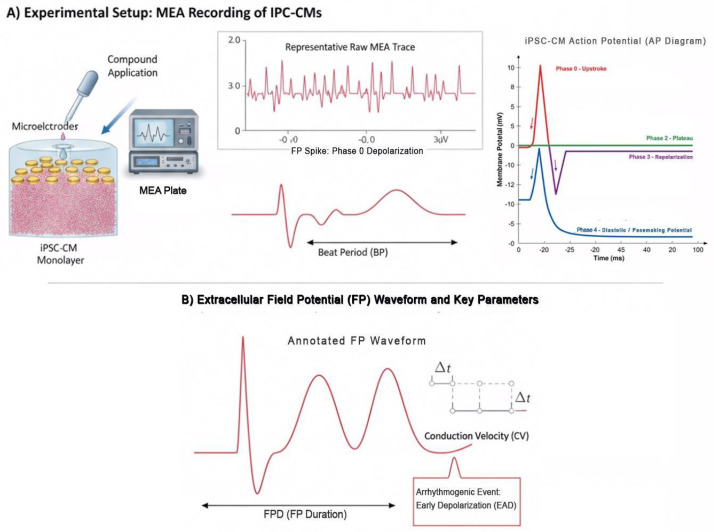
Electrophysiological Profiling of iPSC-CMs Using MEA. MEA profiling of iPSC-CMs links extracellular field potential changes to specific ion channels, serving as a platform for arrhythmogenic risk assessment and drug screening. (**A**) Experimental Setup: MEA Recording of IPC-CMs. This section details the method for recording electrical activity from iPSC-CMs using a Microelectrode Array (MEA) system. iPSC-CM Monolayer: A layer of iPSC-CMs cultured on the MEA plate. Microelectrodes: Tiny electrodes embedded in the MEA plate that detect electrical signals from the cells. Compound (Drug/Vehicle) Application: The process of adding a test compound or a control vehicle to the iPSC-CMs. MEA System & Amplifier: The equipment that records and amplifies the electrical signals detected by the microelectrodes. Representative Raw MEA Trace: An example of the raw electrical activity recorded, showing field potentials (FP spikes). Beat Period (BP): The time interval between consecutive field potentials, indicating the heart rate of the iPSC-CMs. iPSC-CM Action Potential (AP) Diagram: A schematic of the typical iPSC-CM action potential, highlighting key ion currents responsible for different phases: Red line: Phase 0/Upstroke with INa (Nav1.5): Sodium current, responsible for the rapid depolarization phase. Phase 1, mediated by the transient outward K+ current (I_t0_), is responsible for the rapid, brief drop in potential immediately following Phase 0. It is often minimal or absent in immature iPSC-CMs. Green line: Phase 2: ICa,L (Cav1.2): L-type calcium current, contributing to the plateau phase. Purple Line: Phase 3: Represents prolonged, stable positive potential. IKr (hERG): Rapid delayed rectifier potassium current, crucial for repolarization. Blue line: Diastolic/Pacemaking Potential. IK1 (Kir2.1): Inward rectifier potassium current, maintaining resting membrane potential. If (HCN4): Funny current, contributing to spontaneous depolarization. (**B**) Extracellular Field Potential (FP) Waveform and Key Parameters. This section explains how to analyze the field potentials and link them to ion channel mechanisms and arrhythmia risk. Annotated Field Potential (FP Waveform): A detailed view of a single field potential, showing: Conduction Repolarization (CV): The speed at which electrical signals propagate. FPD (FP Duration: Repolarization Reserve): The duration of the field potential, indicating the time it takes for the cells to repolarize. Δt indicates the duration measurement. EADs (Early Afterdepolarizations): Abnormal depolarizations occurring during repolarization, indicative of arrhythmia risk. Ion Channel Mechanisms & Arrhythmia Risk: A table correlating changes in FP findings with affected ion channels and associated arrhythmia risks: FPD Prolongation: Linked to reduced IKr current, increasing TdP (Torsades de Pointes) risk. FPD Prolongation with EADs: Indicates potential issues with IKr, ICa,L, and TdP risk. Spike Amplitude/Conduction Block/Re-entry: Reduced spike amplitude or conduction block can lead to re-entry arrhythmias. iPSC-CM Immunity Limitations: Notes on inherent characteristics of iPSC-CMs that might affect drug screening: reduced I_K1 & persistent I_If lead to depolarized RMP (resting membrane potential), spontaneous beating, and baseline proarrhythmia. In summary, MEA profiling of iPSC-CMs links external field potential changes to specific ion serving as a platform for aloe risk assessment and drug screening.

MEA recordings (Figure 1) quantify how immature electrophysiology translates into slowed conduction and depolarized resting potentials, supporting the observed arrhythmic risk in vitro. Consistent with our findings in mitochondria-enriched extracellular vesicle studies [1,16], MEA profiles reveal that energy insufficiency exacerbates APD variability and triggers delayed afterdepolarizations, emphasizing the convergence of bioenergetics and electrophysiology in arrhythmogenesis.

Electrophysiologically, immature iPSC-CMs display slower conduction velocities, reduced sodium current density, and incomplete repolarization capacity [1,3,5]. These features prolong action potential propagation, increase dispersion of refractoriness, and predispose to conduction block [74]. Importantly, immaturity not only predisposes to arrhythmias in vitro, but also compromises their integration when transplanted into mature host myocardium. Transplanted cells with depolarized resting potentials can generate ectopic pacemaking activity, while mismatch in gap junctional coupling (e.g., reduced connexin 43 expression) impairs synchronous contraction and may anchor reentrant arrhythmias [75,76,77,78].

**Figure 2:**
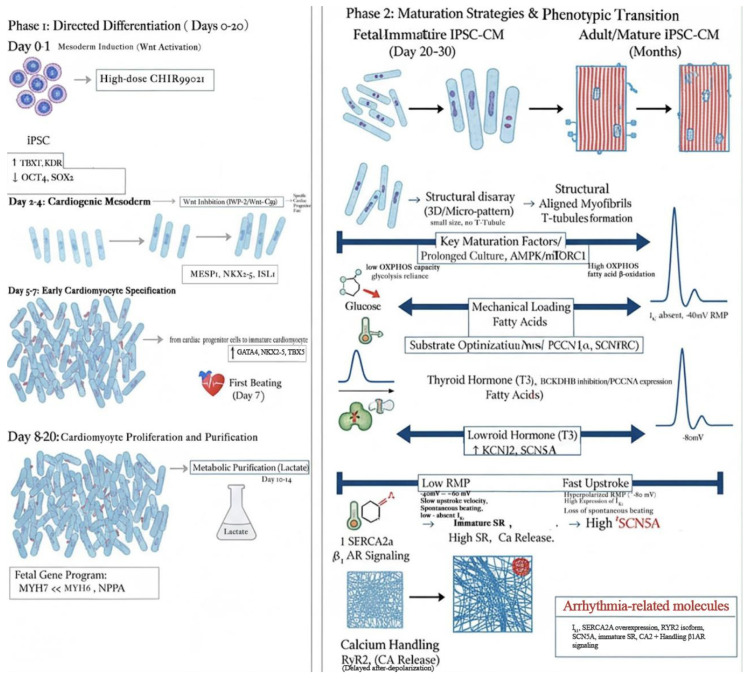
Biomolecular Timeline of iPSC-CM Differentiation and Maturation. This timeline visually charts the precise, directed developmental progression of iPSC-CMs. The process demands critical balance and timed synergy between opposing molecular forces to achieve its ultimate goal: a functional adult-like heart cell. The initial Differentiation Phase (Days 0–20) is a rapid, controlled sequence of events. It begins with a powerful push to activate Wnt signaling (CHIR99021) to establish the general mesoderm. This high-force action is immediately met with an equal and opposite force—Wnt inhibition (IWP-2)—to perfectly balance the signal, thereby ensuring fate commitment to the heart lineage. This balance is necessary to trigger the emergence of the core cardiac transcription factors (GATA4, NKX2-5, TBX5). These factors then work in cooperative partnership to build the foundational structure and function of the embryonic heart cell. The following Maturation Phase (Day 20+ to Months) represents the cell’s mission toward a higher, adult-level standard—a quest for physiological perfection. The cell must fundamentally transform by abandoning its immature, fetal-like nature (glycolytic metabolism, low IK1 expression, disorganized structure) and embrace a vastly expanded set of adult traits. This maturation involves a visionary overhaul that includes: 1. Metabolic Redirection: A crucial shift from sugar-based glycolysis to \text{fatty acid oxidation} (OXPHOS) to meet the adult heart’s massive energetic demands. 2. Electrophysiological Stabilization: Harmonious restructuring of ion channel expression, most importantly the significant upregulation of IK (KCNJ2) to stabilize the resting membrane potential. 3. Structural Organization: Achieving mature sarcomere alignment and calcium handling capacity (RyR2, SERCA2a) through external biophysical and hormonal cues (T3, mechanical load). Failure to complete this maturation, particularly the lack of IK1 and RyR2/SERCA2a maturation, results in the cell being stuck at the immature, arrhythmogenic state. The entire timeline is, therefore, a precise, sequential search for functional equilibrium to fulfill its destiny as a mature, working cardiomyocyte.

Recent strategies have attempted to overcome immaturity through mechanical loading, 3D engineered tissues, electrical pacing, and metabolic reprogramming toward fatty acid oxidation [2,3,5]. Nonetheless, no single approach has fully recapitulated adult-like structure-function coupling. This remains the central translational barrier (Figure 2).

### 3.4. Integrative Perspective

From an integrative biomolecular standpoint, the arrhythmogenic risk of iPSC-CMs emerges not from isolated defects but from the convergence of three interlinked phenomena: electrophysiological immaturity, subtype heterogeneity, and metabolic underdevelopment [1,3,5]. These limitations intertwine, as impaired mitochondrial function amplifies ionic instability [5], and heterogeneity exacerbates conduction dispersion [3,5,79,80]. This echoes a recurring theme across our publication trajectory: regenerative solutions must address not only cellular survival but also electrical fidelity and energetic competence.

Thus, to advance iPSC-CMs toward safe clinical translation, therapeutic strategies must be systemically multipronged—combining bioengineering for subtype specification, metabolic maturation for energetic stability, and precision gene editing to normalize electrophysiological substrates. Only by simultaneously targeting these converging axes can the field mitigate proarrhythmic risk and unlock the full regenerative promise of iPSC-CMs.

The complexity of arrhythmogenic risk in iPSC-CMs is not merely theoretical—it manifests in observable model-specific phenotypes. Table 2 presents a curated overview of 3D iPSC-CM systems, integrating electrophysiology, cellular heterogeneity, and bioenergetic maturation. This synthesis aligns with our prior studies demonstrating how mitochondrial dynamics and extracellular vesicle interventions intersect with arrhythmic vulnerability [1,5,15,16].

Building on these mechanistic insights and model-specific observations, the next section explores strategies to mitigate arrhythmogenic risk through targeted maturation, gene editing, co-culture, and pharmacological interventions.

## 4. Therapeutic Strategies to Mitigate Arrhythmogenic Risks

**Figure 3:**
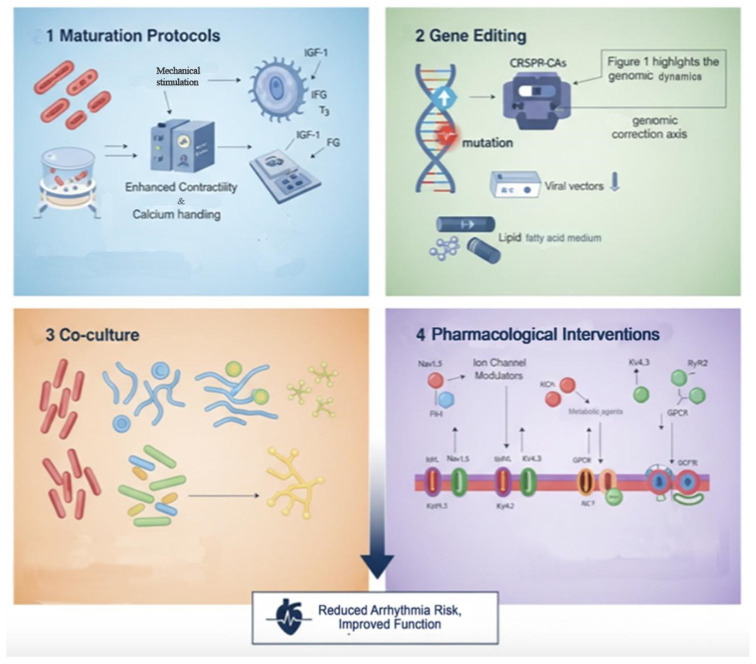
Therapeutic Strategies to Mitigate Arrhythmogenic Risks in iPSC-CMs. This outlines current approaches to enhance the safety and functionality of iPSC-CMs for therapeutic applications. **1.** Maturation Protocols, top-left panel illustrates techniques such as optimized extracellular matrix components, mechanical stimulation, and hormonal treatments (e.g., IGF-1, thyroid hormone) to promote structural and functional maturation, leading to improved contractility and calcium handling, which are critical for reducing arrhythmogenic risk. **2.** Gene Editing (highlights the genomic correction axis): Here, advanced gene editing technologies like CRISPR-Cas systems are depicted, offering precise correction of genetic mutations linked to arrhythmogenic conditions. Viral and non-viral delivery methods for these genetic tools are also shown. **3.** Co-culture (Multicellular niche strategies summarized): This panel presents the benefits of co-culturing iPSC-CMs with other cell types (e.g., fibroblasts, endothelial cells). These multicellular niches mimic the native heart environment, promoting electrical coupling, paracrine signaling, and overall tissue organization, thereby stabilizing cardiac rhythm. **4.** Pharmacological Interventions (modulation): This section details the use of small molecules to modulate specific ion channels (e.g., NaV1.5, KV4.3) and metabolic pathways. These interventions aim to correct electrical imbalances and metabolic deficiencies that contribute to arrhythmogenesis in iPSC-CMs. Collectively, these strategies converge to achieve the overarching goal of reducing arrhythmia risk and improving the overall function of iPSC-CMs for regenerative medicine and disease modeling.

The translational utility of iPSC-CMs is significantly constrained by their inherent arrhythmogenicity [1,3]. Building on the electrophysiological immaturity, cellular heterogeneity, and susceptibility to aberrant conduction highlighted earlier, diverse therapeutic strategies have been designed to attenuate these risks. These interventions are neither unidimensional nor merely corrective; they seek to re-engineer the fundamental structural and functional deficits of iPSC-CMs in ways that align with both clinical safety and the mechanistic elegance of cardiac electrophysiology. Four principal domains—maturation protocols, gene editing, co-culture systems, and pharmacological interventions—emerge as the most promising avenues. Figure 3 illustrates these therapeutic strategies and their proposed mechanisms to mitigate arrhythmogenic risk in iPSC-CMs.

### 4.1. Maturation Protocols

One of the most direct strategies to mitigate arrhythmogenic risks in iPSC-CMs is to accelerate and enforce their structural and electrophysiological maturation [1,3]. Immaturity manifests in depolarized resting membrane potentials, prolonged action potential durations, and aberrant calcium handling [3]—all risk factors for triggered activity and reentrant arrhythmias [1,3]. To address this, electrical pacing protocols have been widely adopted. Studies have demonstrated that chronic field stimulation promotes alignment of sarcomeres, improves gap junction connectivity, and induces adult-like action potential profiles [1,2,3,5,98,99,100,101]. Importantly, long-term pacing leads to the development of transverse tubule networks [102], and improved calcium transient synchrony, reducing early afterdepolarizations [1,2,3,5,102,103].

Most maturation studies remain at the in vitro stage, conducted in monolayer or engineered tissue formats. However, a few in vivo validations have emerged for example, preconditioned iPSC-CM grafts with electrical pacing or mechanical stimulation show improved survival and reduced ectopy in rodent and myocardial infarction models [102,103,104]. These findings underscore the translational feasibility of physiologically matured grafts although full electrophysiological equivalence to adult myocardium has not yet been achieved.

Biochemical conditioning further reinforces this maturation trajectory. Hormonal cues such as triiodothyronine (T3) and glucocorticoids [1,3,5], as well as metabolic interventions shifting cells from glycolysis toward oxidative phosphorylation [5], have been shown to refine electrophysiological phenotypes. Fatty acid supplementation, for instance, enhances mitochondrial density and energetics, thereby reducing delayed afterdepolarizations [1,5]. Integration of mechanical stress, through engineered heart tissues or micropatterned substrates, complements these interventions by enforcing physiological force–length relationships and gap junction localization [104,105].

Collectively, these strategies represent substantive methodological advances aimed at reproducing the electrophysiological characteristics of the mature myocardium, thereby reducing arrhythmogenic propensity by addressing its developmental origins.

### 4.2. Gene Editing Techniques

While maturation addresses phenotypic immaturity, arrhythmogenicity can also stem from inherited or acquired channelopathies. Here, gene editing represents an unparalleled corrective approach. CRISPR/Cas9 [1,2,3,5] and related nucleases have demonstrated precision in repairing pathogenic mutations in genes such as SCN5A (encoding the cardiac sodium channel Na_v1.5) [2,3,5] and KCNH2 (encoding the hERG potassium channel), which are frequently implicated in long QT syndrome [9,106]. Successful correction restores normal action potential repolarization and dramatically reduces the frequency of arrhythmic events in vitro.

Beyond single-gene corrections, CRISPR-based transcriptional regulators offer opportunities for polygenic modulation. For example, targeted upregulation of Kir2.1 inward rectifier potassium channels has been shown to hyperpolarize resting membrane potentials and suppress automaticity in iPSC-CMs [107]. Emerging base-editing and prime-editing tools further promise reduced off-target effects [107,108], which is critical for clinical translation.

To date, most gene-editing approaches have demonstrated preclinical feasibility in vitro or in small animal disease models. For example, CRISPR correction of SCN5A and KCNH2 mutations in iPSC-CMs has restored normal electrophysiological function in murine xenografts, reducing arrhythmic episodes post translation [93,109]. However, no human clinical trials have implemented gene-edited cardiomyocyte transplantation, emphasizing that this domain remains in the translational research phase rather than clinical validation.

Thus, gene editing operates at the genomic substratum of arrhythmogenesis, transforming iPSC-CMs from inherently unstable models into precision-tailored systems aligned with patient-specific therapy.

### 4.3. Co-Culture Systems

Arrhythmogenicity in iPSC-CMs also arises from their isolation from the multicellular, heterogeneous context of native myocardium. Co-culture systems directly address this by restoring cellular cross-talk. Incorporation of cardiac fibroblasts, endothelial cells, and smooth muscle cells has been shown to enhance electrical conduction and reduce dispersion of repolarization [109]. Endothelial cells, through paracrine secretion of neuregulin-1 and VEGF [1,3,5], induce electrophysiological remodeling that stabilizes action potential propagation [110].

Fibroblast co-culture improves conduction velocity by depositing extracellular matrix proteins that promote anisotropic conduction pathways. Similarly, epicardial-derived cells accelerate structural maturation, reducing calcium alternans and arrhythmic triggers. These strategies are further strengthened in three-dimensional engineered tissues, where multicellular integration creates physiologically relevant conduction patterns.

These co-culture and tissue-engineered constructs have demonstrated functional integration and improved conduction in preclinical small-animal models, but long-term in vivo validation—particularly for graft-host electrical coupling and immunological compatibility—remains limited. Thus, co-culture systems represent a translationally promising but still preclinical platform.

Through co-culture, iPSC-CMs are no longer insulated entities with incomplete maturation, but participants in a reconstructed myocardial niche, wherein arrhythmogenic vulnerability is minimized by the collective buffering of diverse cardiac lineages.

### 4.4. Pharmacological Interventions

Pharmacological strategies represent the most immediately translatable interventions to reduce arrhythmogenicity. Ion channel modulators, such as late sodium current inhibitors (ranolazine) [111,112,113,114] and I_Kr activators [115], have been employed to shorten action potential durations and suppress afterdepolarizations. Calcium handling abnormalities, a key source of delayed afterdepolarizations, can be mitigated using SERCA2a activators and ryanodine receptor stabilizers [3,5,7,116], which restore intracellular calcium homeostasis.

More recently, repurposing of antiarrhythmic drugs has been investigated in patient-derived iPSC-CMs carrying pathogenic mutations [117]. For example, flecainide [117,118] and mexiletine [119,120,121,122] have successfully normalized arrhythmic phenotypes in long QT and catecholaminergic polymorphic ventricular tachycardia models. While these approaches do not correct underlying immaturity, they provide critical safety margins for translational applications, particularly in drug testing platforms.

Unlike the other modalities, pharmacological interventions possess partial clinical validation, as many of the compounds used (e.g., ranolazine, flecainide, mexiletine) are already approved antiarrhythmic agents repurposed for testing iniPSC_CM models. This confers an immediate translational advantage. However, systematic in vivo evaluation of these drugs’ safety and efficacy in grafted iPSC-CMs is still sparse, necessitating targeted studies in large-animal cardiac models.

The challenge lies in balancing efficacy with proarrhythmic liability—underscoring the importance of combining pharmacological modulation with maturation, gene editing, and co-culture strategies.

### 4.5. Integrative Outlook

The mitigation of arrhythmogenic risk in iPSC-CMs is not achieved through a single approach, but through an integrated interplay of maturation enforcement, genomic correction, multicellular reconstruction, and pharmacological modulation. Each intervention targets a distinct axis of vulnerability, and only their convergence can approximate the electrophysiological fidelity necessary for reliable preclinical and therapeutic application. In this context, therapeutic innovation reflects a paradigm grounded in precision, rigor, and a comprehensive understanding of the heart’s electrophysiological complexity.

Taken together, the maturity gradient across these approaches is evident: Maturation and gene-editing strategies are advancing through preclinical validation, co-culture systems occupy the translational interface, and pharmacological approaches already have partial clinical evidence. Clear delineation of these stages is essential for designing phased translational pipelines and aligning regulatory expectations for future clinical trials.

## 5. Discussion

### 5.1. Integration of Findings

The current synthesis highlights how arrhythmogenic risk in iPSC-derived cardiomyocytes arises from a multifactorial convergence of electrophysiological immaturity, cellular heterogeneity, and incomplete structural–metabolic development. Each of these deficits functions as a destabilizing node within the broader bioelectric network: depolarized resting potentials and deficient IK1 currents amplify spontaneous activity, heterogeneity in subtypes and conduction properties introduces dispersion, and immature mitochondrial energetics exacerbate triggered activity through impaired calcium buffering. The therapeutic strategies under review—maturation protocols, gene editing, co-culture, and pharmacological interventions—address these risks in complementary ways, but no single approach neutralizes all axes of vulnerability.

As highlighted in Table 3, structural, ionic, and metabolic immaturity in iPSC-CMs underpins the arrhythmogenic substrate. Maturation protocols aim to reconcile these deficits, guiding the cells toward adult-like electrophysiological profiles while stabilizing bioenergetics [1,5,16].

What emerges is a systems-level principle: the arrhythmogenic phenotype is not the result of one defective component, but of maladaptive interactions across molecular, electrophysiological, and structural domains. This echoes a theme that has surfaced in our own recent publications on extracellular vesicle–mediated mitochondrial modulation [15,16], where the cross-talk between energetics and electrophysiology dictates cell survival and fidelity. Just as mitochondrial EVs restore bioenergetic competence by targeting multiple checkpoints simultaneously, the future of iPSC-CM stabilization lies in multipronged, convergent interventions.

Maturation protocols move the cells closer to adult-like states by enforcing bioelectric discipline and metabolic balance. Gene editing provides corrective precision at the genomic substrate, ensuring that even disease-specific models can regain stability. Co-culture systems recreate the multicellular dialog of native myocardium, while pharmacological modulation supplies clinically translatable buffers against acute arrhythmic triggers. Taken together, these strategies can be conceptualized as a four-tiered scaffold, where structural maturation, genetic stability, paracrine support, and drug-based fine-tuning act in synergy to stabilize the iPSC-CM phenotype.

### 5.2. Clinical Implications

From a translational perspective, the dual role of iPSC-CMs—as both disease models and potential regenerative grafts—necessitates careful parsing of their arrhythmogenic risk. In preclinical pharmacology, iPSC-CMs offer unparalleled opportunities for patient-specific modeling of channelopathies and drug testing. However, the very electrophysiological variability that enriches disease modeling can obscure predictive fidelity if not adequately standardized. Regulatory authorities, including the FDA and EMA, have underscored the need for GMP-compatible production pipelines, uniform quality control metrics, and validated electrophysiological benchmarks to ensure safety in both disease modeling and therapy [1,3,5,16].

In regenerative contexts, the arrhythmogenic liabilities are magnified. Transplantation of immature or heterogeneous iPSC-CMs into injured myocardium risks creating ectopic pacemaking foci, conduction block, or reentrant substrates. Clinical trials in large-animal models have shown both the promise and peril of this approach: engrafted cells can contribute to force generation, but they also induce ventricular arrhythmias if structural and electrophysiological mismatch persists. To safely harness iPSC-CMs, clinical translation will require the integration of maturation protocols prior to transplantation, gene-edited stabilization of electrophysiological properties, and incorporation of co-culture-derived support cells to promote synchronized coupling.

Moreover, pharmacological adjuncts could play a transitional role in early-stage graft survival, serving as arrhythmia suppressors until transplanted cells achieve greater maturity. This layered therapeutic design reflects a pragmatic yet revolutionary shift: moving from cell replacement as a singular intervention toward a combinatorial therapy, where engineering, molecular correction, and pharmacological regulation are integrated into one cohesive platform.

### 5.3. Future Directions

Future research must now advance beyond proof-of-principle interventions toward scalable, clinically aligned solutions. Several avenues merit emphasis.

1. Standardization of Maturation Platforms: Electrical pacing, metabolic reprogramming, and 3D tissue engineering have each shown partial success. The next frontier lies in combining these into unified, GMP-compliant bioreactor systems capable of producing mature, arrhythmia-resistant iPSC-CMs at scale. Longitudinal studies should evaluate not only electrophysiological endpoints but also the durability of maturation post-transplantation [2,3].
2. Next-Generation Gene Editing: While CRISPR/Cas9 correction has established feasibility, the emergence of prime editing and epigenetic reprogramming offers the possibility of correcting polygenic arrhythmogenic substrates with reduced off-target risk [155,156,157]. Pairing gene editing with real-time functional readouts, such as optical mapping of conduction and calcium transients, could provide a closed-loop framework for tailoring therapies at the single-cell level [158,159,160,161,162].
3. Bioengineered Multicellular Niches: Co-culture approaches need to evolve into fully bioengineered myocardial constructs, where iPSC-CMs are integrated with fibroblasts, endothelial cells, and autonomic inputs in 3D microenvironments that replicate the physiological conduction hierarchy. Integration of vascularization strategies—such as endothelialized scaffolds or angiogenic extracellular vesicles—may further reduce arrhythmic substrates by optimizing oxygen and nutrient supply [2,3,5,15].
4. Pharmacological-Genetic Hybrids: There is untapped potential in designing therapies that combine transient pharmacological stabilization with long-term genomic correction. For instance, patients receiving iPSC-CM grafts may initially be treated with ion channel modulators or calcium stabilizers until gene-edited, matured grafts achieve stable conduction synchrony.
5. Integration with Bioenergetics Therapies: Our previous findings on mitochondria-enriched extracellular vesicles underscore how metabolic integrity underpins electrical stability. A critical research direction is the co-application of metabolic modulators—whether vesicle-based, small-molecule, or gene-driven—alongside iPSC-CM transplantation. By restoring mitochondrial architecture and oxidative phosphorylation, one can reduce delayed afterdepolarizations and stabilize excitation–contraction coupling.

#### Unresolved Challenges and Limitations

Despite rapid progress, several fundamental challenges remain unresolved. Immunogenicity continues to pose a significant translational barrier, even in autologous iPSC applications, as reprogramming-induced mutations, incomplete epigenetic resetting, or residual undifferentiated cells can provoke immune recognition. The development of hypoimmunogenic or HLA-engineered iPSC lines has mitigated, but not eliminated, this risk. Scalability also limits clinical deployment: current maturation and purification workflows rely heavily on labor-intensive protocols, variable reagent quality, and non-standardized culture systems that hinder reproducibility and GMP alignment. Automated, closed-system bioreactors and chemically defined media formulations are urgently needed to ensure consistent, high-volume production of electrophysiologically mature cardiomyocytes.

Equally critical is long-term integration after transplantation. Preclinical models show that engrafted iPSC-derived cardiomyocytes often exhibit incomplete electrical coupling with host myocardium, creating conduction discontinuities that favor reentry or ectopic activity. Addressing this will require innovations in bioengineered scaffolds, synchronized pacing regimens, and vascularization strategies that promote structural and metabolic assimilation. Finally, long-term safety monitoring—particularly for arrhythmia, tumorigenicity, and graft attrition—remains essential before large-scale trials can be ethically justified. Acknowledging these translational constraints ensures that future therapeutic frameworks evolve not only through technological sophistication but also through regulatory, ethical, and manufacturing rigor.

### 5.4. Concluding Perspective

The arrhythmogenic risk of iPSC-CMs reflects both the beauty and burden of cellular plasticity: these cells retain developmental openness that allows disease modeling and regenerative flexibility, yet this same immaturity makes them electrically unstable. Therapeutic innovation must therefore proceed with both respect for this complexity and obsession with precise correction. By weaving together bioengineering, molecular editing, multicellular integration, and pharmacological support, the field is poised to evolve from incremental optimization into systemic, revolutionary solutions.

The challenge—and the opportunity—lies not merely in making iPSC-CMs “safer,” but in designing them as intelligent therapeutic units that honor the physiological elegance of the human heart.

## 6. Conclusions

iPSC-CMs represent an indispensable model for cardiovascular research and regenerative medicine, yet their utility is significantly constrained by arrhythmogenic risks. These risks arise from electrophysiological immaturity, ion channel dysregulation, and conduction heterogeneity, all of which compromise their predictive validity for human cardiac physiology and their translational safety for therapy. Key findings underscore that the immature action potential morphology, aberrant calcium handling, and inconsistent gap junction coupling collectively amplify susceptibility to pro-arrhythmic events. Additionally, variability among differentiation batches and across laboratories remains a persistent challenge, impeding reproducibility and standardization.

On the therapeutic front, promising strategies are emerging. Maturation protocols—including long-term culture, electrical pacing, mechanical loading, and metabolic reprogramming—have demonstrated substantial potential in enhancing structural and functional alignment of iPSC-CMs with adult phenotypes. Gene editing technologies such as CRISPR/Cas9 offer precision correction of pathogenic mutations, while co-culture systems incorporating fibroblasts, endothelial cells, and cardiac progenitors have improved electrophysiological synchrony. Pharmacological interventions targeting ion channels or metabolic regulators are being actively explored to stabilize conduction and mitigate arrhythmogenicity. Collectively, these strategies illustrate a dynamic, multi-pronged approach toward ensuring both safety and efficacy.

### 6.1. Recommendations

For researchers, several best practices are recommended. First, standardized protocols for differentiation, maturation, and electrophysiological assessment should be prioritized to minimize batch-to-batch variability. Integration of multimodal assays—patch-clamp, optical mapping, and single-cell transcriptomics—will enable more comprehensive evaluation of arrhythmic risk. Second, therapeutic interventions should be systematically tested in both reductionist (single-cell) and integrated (engineered tissue, organoid, and in vivo) models to better capture emergent properties of cardiac networks. For clinicians, cautious interpretation of iPSC-CM–based preclinical findings is warranted until validated benchmarks for maturity and arrhythmic safety are universally established. Clinical translation should proceed only when supported by robust preclinical pipelines that incorporate long-term safety monitoring.

### 6.2. Final Thoughts

The trajectory of iPSC-CM research embodies a dual imperative: to harness their extraordinary potential for regenerative cardiology while rigorously addressing their intrinsic limitations. Arrhythmogenic risks, though formidable, are not insurmountable. Instead, they represent a scientific frontier that compels the integration of bioengineering, molecular genetics, and pharmacological innovation. By embracing multidisciplinary strategies and fostering collaborative frameworks, the field can progress toward safe, reproducible, and clinically impactful applications. Ultimately, sustained inquiry into both fundamental mechanisms and translational methodologies will determine whether iPSC-CMs can fulfill their promise—not only as experimental models but also as therapeutic agents capable of transforming the management of cardiovascular disease.

## Figures and Tables

**Table 1:** Overview of published 2D iPSC-CM Cardiac Arrhythmia Disease Models.

Disease Model	Primary Genetic/Pathogenic Mechanism	Representative Phenotype (Electrophysiological & Metabolic)	Interventions & Remaining Limitations	References
Long QT Syndrome (LQTS) *	Mutations in KCNQ1, KCNH2, SCN5A causing impaired IKs, IKr, or enhancing late INa	Prolonged APD, early afterdepolarizations, exaggerated β-adrenergic response; elevated ROS, glycolytic bias	Mexiletine or CRISPR correction shorten APD; immaturity of IK1 and Ca^2+^ cycling persists	[1,34,35,36,37,38]
Catecholaminergic Polymorphic Ventricular Tachycardia (CPVT)	RYR2, CASQ2 mutations destabilizing SR Ca^2+^ release	Triggered activity and delayed afterdepolarizations; fragmented mitochondria, low ATP/ADP ratio	Flecainide restores Ca^2+^ stability; incomplete excitation–energy coupling remains	[2,29]
Brugada Syndrome	SCN5A loss-of-function reducing INa	Slowed conduction and conduction block; mitochondrial depolarization under stress	Sodium current enhancers normalize upstroke; gap junction immaturity promotes reentry	[13,39,40]
Arrhythmogenic Right Ventricular Cardiomyopathy (ARVC)	PKP2, DSP, DSG2 mutations impairing desmosomes	Reduced adhesion, slowed conduction, Ca^2+^ wave heterogeneity; lipid accumulation	PPAR/Wnt modulation lowers lipogenesis; structural syncytium loss in 2D persists	[3,41,42]
Hypertrophic Cardiomyopathy (HCM)	MYH7, MYBPC3 mutations altering sarcomeric contractility	Prolonged APD, Ca^2+^ alternans, oxidative stress; hyperfused mitochondria	Antioxidants reduce EADs; lack of chronic mechanical conditioning remains	[17,43,44,45]
Dilated Cardiomyopathy (DCM)	TTN, LMNA, RBM20 defects weakening sarcomeres	Slowed conduction, prolonged Ca^2+^ decay; reduced mitochondrial mass, ATP deficit	Gene correction and T3 maturation improve APs; contractile recovery incomplete	[17,46,47]
Drug-Induced QT Models	Pharmacological IKr or INa blockade	Dose-dependent APD prolongation, Ca^2+^ instability, ROS accumulation	Ranolazine reverses QT prolongation; maturity variability limits predictivity	[48,49,50]
Mitochondrial Cardiomyopathies	POLG, mt-tRNA mutations impairing respiration	Depolarized mitochondria, reduced Ca^2+^ uptake, EADs/DADs	Mitochondria-enriched EVs restore stability; mtDNA heteroplasmy not modeled	[15,16,51,52,53]
Metabolic Arrhythmia (Diabetic/Stress)	Hyperglycemia, lipotoxicity, oxidative injury	APD variability, Ca^2+^ leak, ROS-driven triggered activity	Antioxidants and EV rescue reduce arrhythmia; chronic stress effects untested	[16,54,55,56,57]
Ischemia/Reperfusion Injury	Hypoxia–reoxygenation injury	Afterdepolarizations, Ca^2+^ alternans, ATP depletion	EV-based mitochondrial transfer restores stability; lack of microvascular coupling persists	[2,3,5]

* iPSC-CMs recapitulate key electrophysiological phenotypes (e.g., prolonged APD, EADs, DADs, conduction defects) and related mitochondrial/metabolic disturbances found in patients. Key limitations across models include the functional and structural immaturity of iPSC-CMs (e.g., immature IK1, Ca^2+^} handling) and the simplified nature of 2D culture, which lacks physiological mechanical load, cell-matrix interaction, and the complex tissue structure seen in vivo. Despite limitations, these models are valuable for identifying novel pharmacological and genetic interventions, facilitating drug discovery, and advancing the understanding of arrhythmogenesis.

**Table 2:** Overview of published 3D iPSC-CM Arrhythmia Models.

Study/Model	Cell Source& Subtype Composition	3D Platform/Scaffold	Electrophysiology Highlights	Arrhythmogenic Observations	Mitigation Strategies
Fassina et al. (2022) [81]	iPSC-derived ventricular, atrial, nodal-like cells (mixed)	Engineered heart tissue (EHT)	Spontaneous automaticity; prolonged APD; low IK1	Early afterdepolarizations (EADs); beat-to-beat variability	Electrical pacing, T3 hormone supplementation
Lemme et al. (2019) [82]	Ventricular-biased iPSC-CMs	Biomimetic mechanical laid + 3D EHT	Increased conduction velocity; improved Ca^2+^ handling	Reduced DADs, but minor reentry circuits persisted	Chronic electrical pacing, mechanical stretch, metabolic shift
Xu et al. (2022) [83] & Seguret et al. (2024) [84]	Mixed ventricular-atrial iPSC-CMs	3D ring-shaped microtissues	Action potential heterogeneity; slow conduction	Reentry-like propagation in ring model	Micro-patterned substrate alignment
Andrée et al. (2024) [85] and Vanderslice et al. (2024) [86]	Patient-specific iPSC-CMs (Long QT)	Fibrin-based 3D tissues	Prolonged APD; arrhythmic Ca^2+^ transients under adrenergic stimulation	Catecholaminergic polymorphic ventricular tachycardia (CPVT)-like events	β-adrenergic blockers, CRISPR correction of KCNH2
Li et al. (2018) [87] and Goldfracht et al. (2020) [88]	Ventricular iPSC-CM	Hydrogel-embedded 3D EHT	Improved conduction velocity with cell alignment	Reduced spontaneous arrhythmias	Electrical stimulation + fatty acid metabolic maturation
Ikeda et al. (2021) [89]	Heterogenous MSC/iPSC-CMs (Atrial, ventricular, nodal)	3D scaffold + mitochondria-enriched EV supplementation	Normalized APD, improved Ca^2+^ transient synchronization	DADs reduced; conduction dispersion minimized	EV-mediated metabolic enhancement, electrical pacing, subtype alignment
Gartner (2022) [90] and Tadano (2021) [91]	Ventricular iPSC-CMs	Engineered cardiac tissues with fibroblast/endothelial co-culture	Enhanced APD uniformity; improved conduction	Minor ectopic activity; lower incidence of reentry	Co-culture with fibroblasts and endothelial cells; extracellular matrix optimization
Campostrini et al. (2023) [92], Liang et al. (2016) [93], and Lemoine et al. (2017) [94]	iPSC-CMs with SCN5A mutation	3D EHT	Slow Na^+^ current; conduction velocity deficit	Ectopic pacemaking; triggered activity	CRISPR correction; pharmacological sodium channel modulators
Pourchet et al. (2025) [95], Esser et al. (2023) [96], and Bliley (2022) [97]	Ventricular iPSC-CMs	3D bioprinted tissues with microvascular perfusion	Reduced APD variability; stable Ca^2+^ transients	Minimal spontaneous arrhythmias	Perfusion-enhanced metabolic maturation; mechanical and electrical cues

**Table 3:** Electrophysiological Properties comparison: iPSC-CMs vs. Adult Cardiomyocytes.

Parameter	iPSC-CMs	Adult Ventricular CMs	Functional Consequences/Clinical Implication	References
Resting Membrane Potential (RMP)	Depolarized (−50 to −65 mV) due to low IK1 density (↓ KCNJ2 expression)	Stable (−80 to −90 mV) via robust IK1 conductance	Depolarized RMP increases automaticity and ectopic firing	[49,123,124,125,126,127]
Action Potential Duration (APD)	Prolonged and variable (200–500 ms); dependent on immature IKs and IKr	Stable, shorter APD (150–250 ms)	Promotes early afterdepolarizations (EADs) and QT prolongation	[128,129,130,131]
Automaticity/Pacemaker Activity	Spontaneous beating via persistent funny current (↑ HCN4)	Quiescent without sinoatrial input	Uncontrolled pacemaking contributes to ectopic rhythm generation post-transplant	[2,21,22,132,133]
Sodium Current (INa)	Reduced peak INa density; slow upstroke velocity (V_max_ ↓)	High amplitude INa ensures rapid depolarization	Slower conduction velocity, higher conduction block risk	[2,3,5,94,134,135,136]
Inward Rectifier K^+^ Current (IK1)	Severely diminished or absent	Prominent, stabilizes RMP	Destabilized RMP → spontaneous depolarization & triggered activity	[16,49,125,137]
Repolarizing K+ Currents (IKr, IKs)	Low expression and incomplete maturation	Well-developed, ensuring phase 3 repolarization	Prolonged APD and increased dispersion of refractoriness	[5,130,138,139,140]
Calcium Handling	Immature SR; ↓ RyR2 and SERCA2a expression, asynchronous Ca^2+^ transients	Mature SR; synchronized Ca^2+^-induced Ca^2+^ release	Delayed afterdepolarizations (DADs), alternans, and instability	[1,3,5,141,142,143,144,145]
Conexxin 43 (Cx43) Expression	Reduced, disorganized gap junctions	Dense, polarized intercalated disks	Impaired coupling and anisotropic conduction → reentry potential	[1,3,5,146,147,148,149]
Metabolic Profile	Glycolytic dominance; low oxidative phosphorylation, fragmented mitochondria	Fatty acid oxidation; dense cristae and efficient ATP delivery	Energetic mismatch promotes Ca^2+^ instability and arrhythmogenic stress	[2,5,16]
Response to β-Adrenergic Stimulation	Exaggerated or erratic chronotropic response; limited inotropy	Physiological HR increase, synchronized contraction	Enhanced adrenergic sensitivity → catecholaminergic arrhythmias	[49,128,150,151,152,153]
Electrical Conduction Velocity	Slower (10–20 cm/s)	Rapid (40–60 cm/s)	Facilitates reentrant circuit formation	[1,2,3,5,128,140,153,154]
Maturation Response to Mechanical/Electrical Cues	Improves with pacing, 3D culture, and metabolic conditioning	Fully Mature, Stable	External conditioning partially restores adult-like AP but not full fidelity	[1,2,3,5,15,16,17]

The arrows (↑, ↓) in the iPSC-CMs column serve as a concise legend to denote the relative expression level or functional density of a specific parameter or ion channel compared to Adult Ventricular CMs. A down arrow (↓) indicates that the parameter (e.g., I_K1_ density, V_max_) is reduced, low, or diminished in induced pluripotent stem cell-derived cardiomyocytes (iPSC-CMs) relative to their mature counterparts. This reduction often signifies an immature or embryonic state. Conversely, an up arrow (↑) means the parameter (e.g., HCN4 expression, which underlies the funny current) is increased or persistent in iPSC-CMs compared to adult cells. For example, the ↓ KCNJ2 expression causes the depolarized resting membrane potential, while the ↑ HCN4 leads to spontaneous automaticity, both contributing to the functional and clinical limitations of iPSC-CMs.

## Data Availability

All data used in this review were extracted from peer-reviewed published articles. No additional unpublished datasets or statistical code were generated.
